# Economic and physical determinants of the global distributions of crop pests and pathogens

**DOI:** 10.1111/nph.12722

**Published:** 2014-02-11

**Authors:** Daniel P Bebber, Timothy Holmes, David Smith, Sarah J Gurr

**Affiliations:** 1 Department of Biosciences, University of ExeterExeter, EX4 4QD, UK; 2 CABI, WallingfordOxfordshire, OX10 8DE, UK; 3 Rothamsted ResearchNorth Wyke, EX20 2SB, UK

**Keywords:** biogeography, biological invasions, crop protection, pest management, plant pathology, species distributions

## Abstract

- Crop pests and pathogens pose a significant and growing threat to food security, but their geographical distributions are poorly understood. We present a global analysis of pest and pathogen distributions, to determine the roles of socioeconomic and biophysical factors in determining pest diversity, controlling for variation in observational capacity among countries.
- Known distributions of 1901 pests and pathogens were obtained from CABI. Linear models were used to partition the variation in pest species per country amongst predictors.
- Reported pest numbers increased with *per capita* gross domestic product (GDP), research expenditure and research capacity, and the influence of economics was greater in micro-organisms than in arthropods. Total crop production and crop diversity were the strongest physical predictors of pest numbers per country, but trade and tourism were insignificant once other factors were controlled. Islands reported more pests than mainland countries, but no latitudinal gradient in species richness was evident.
- Country wealth is likely to be a strong indicator of observational capacity, not just trade flow, as has been interpreted in invasive species studies. If every country had US levels of *per capita* GDP, then 205 ± 9 additional pests per country would be reported, suggesting that enhanced investment in pest observations will reveal the hidden threat of crop pests and pathogens.

## Introduction

Crop pests and pathogens pose a significant threat to global food security (Strange & Scott, 2005; Flood, 2010; Fisher *et al.*, 2012). Around one sixth of the world's agricultural production is lost to destructive organisms annually, with further losses post-harvest (Oerke, 2006; Flood, 2010). Thousands of species, pathovars and genotypes of viruses, bacteria, fungi, oomycetes, nematodes and insects have evolved and spread to plague farmers since the dawn of agriculture, and both natural and anthropogenic dispersal continually introduce pests into new territories (Anderson *et al.*, 2004; Strange & Scott, 2005; Stukenbrock & McDonald, 2008; Bebber *et al.*, 2013). Tracking the movement of pests around the world is a key endeavour in plant protection (Miller *et al.*, 2009). The presence of a particular pest or pathogen in a country is likely to depend upon numerous, sometimes idiosyncratic, biophysical and socioeconomic factors (Waage & Mumford, 2008; Shaw & Osborne, 2011), leading some researchers to conclude that efforts to identify general patterns of distribution and spread are likely to be futile (Shaw & Osborne, 2011). Although the relative importance of different pest dispersal modes at large scales has been investigated (Brown & Hovmøller, 2002; Anderson *et al.*, 2004), a description of the global distributions of pest species and an analysis of potential drivers is lacking.

Comparison with the biogeographical patterns and processes of other species assemblages may be informative. Major global patterns of native (or ‘wild’) species distributions, such as reduced species richness at high latitudes (Hawkins *et al.*, 2003; Willig *et al.*, 2003; Krug *et al.*, 2009) and on small, isolated islands (Whittaker & Fernández-Palacios, 2007), are driven by natural spatiotemporal variation in climate, water and energy availability, habitat area and disturbance regimes, and by processes of evolution, immigration and extinction that determine where species emerge, which suitable environments they colonize, and where they are able to persist (Gaston & Blackburn, 2000). Crop pests, by contrast, are often introduced by human activities such as trade and travel (Anderson *et al.*, 2004; Fisher *et al.*, 2012), as well as by natural dispersal (Brown & Hovmøller, 2002; Anderson *et al.*, 2004) and are, by definition, organisms dependent upon plants that have, themselves, been widely distributed by humans. Crop pests may therefore follow similar distributional patterns to invasive species in general, as invasive species are largely introduced to new regions through human activities such as trade (Westphal *et al.*, 2008; Hulme, 2009; Pyšek *et al.*, 2010; Essl *et al.*, 2011; Seebens *et al.*, 2013). Thus, the extensive biological invasions literature may indicate the patterns and processes governing crop pest distributions.

In contrast to native species, invasive species appear to be more prevalent on islands (Hulme, 2009), although this has been disputed (Westphal *et al.*, 2008; Diez *et al.*, 2009). Outside the tropics, invasive species richness decreases with latitude, but few invasive species appear to have established within the tropics (Sax, 2001). Socioeconomic drivers, particularly measures of wealth, have been recognized recently as an important determinant of the number of invasive species recorded in a country (Taylor & Irwin, 2004; Pyšek *et al.*, 2010; Essl *et al.*, 2011). This is thought to be because country wealth is strongly linked to historical trade, so that countries that have grown rich through trade have also accidentally imported species in agricultural produce, and because activities of wealthy countries such as horticulture and pet-keeping deliberately introduce exotic species (Hulme, 2009; Pyšek *et al.*, 2010; Essl *et al.*, 2011). The role of wealth can be large enough to overshadow biophysical factors such as habitat availability and climate (Pyšek *et al.*, 2010).

Although wealth has been exclusively interpreted as an indicator of increased propagule pressure, another explanation is possible. There exists a strong observational bias in the invasion literature; for example, a recent review found that more than three quarters of studies were conducted in the developed regions of North America, Europe and Australasia (Pyšek *et al.*, 2008). Subsequent investigations of both invasive species in general, and crop pests in particular, have also been largely limited to these regions (Paini *et al.*, 2010; Pyšek *et al.*, 2010; Essl *et al.*, 2011). By contrast, the tropics and developing world have been ignored, probably because of an implicit assumption that knowledge of invasive species assemblages in these regions is poor (but see, for example, Waage *et al.*, 2008). Given that inadequate sampling of tropical biota has also been recognized for native species (Yesson *et al.*, 2007; Boakes *et al.*, 2010; Feeley & Silman, 2011), that economic indicators such as gross domestic product (GDP) are correlated with scientific and technical capacity (Furman *et al.*, 2002) and that wealth generally increases with latitude (Kummu & Varis, 2011), it is reasonable to assume that the role of wealth in predicting numbers of invasive species and pests per country is, at least in part, related to variation in the ability of a given country to detect, identify and report the presence of invasive species. Simply put, it would be surprising if a developing nation such as Uganda had a better understanding of its invasive species burden than a wealthy, technologically advanced nation such as the USA. The competing hypotheses of trade and observational capacity have been termed the ‘biological’ and ‘institutional’ hypotheses, respectively (Waage *et al.*, 2008).

Here, we analyse a comprehensive database of the known distributions of 1901 crop pests in 195 countries, totalling 60 907 observations, and partition the variance in observed pest numbers per country amongst physical, biological and socioeconomic drivers. We attempt to differentiate between the competing hypotheses of trade and observational capacity by comparing the effects of wealth on reports of taxonomic groups of pests likely to vary in their conspicuousness and ease of identification. For example, insects are relatively easy to see and identify, whereas viruses may appear similar to abiotic stress and require molecular diagnostics for identification, and therefore increasing wealth should have a greater influence on virus than insect detection. We then project how changes in countries’ wealth, through economic development, would likely influence the numbers of pests reported, and, if any effects of wealth are actually surrogates for observational capacity, suggest the numbers of pests already present that have not yet been discovered.

## Materials and Methods

Distributional data for 1901 crop pests and pathogens (collectively termed ‘pests’) were obtained with permission from the Plantwise database, compiled by the organization CABI (2013). The Plantwise database comprises geographical data from the CABI Crop Protection Compendium (CPC), and the Distribution Maps of Plant Pests (DMPP) and Distribution Maps of Plant Diseases (DMPD) compiled by CABI and EPPO (Pasiecznik *et al.*, 2005), and other minor sources. Pests are selected for inclusion by phytosanitary experts because of their global or regional economic significance. DMPP and DMPD data have been compiled by computerized search of millions of abstracts in the scientific and grey literature (with consultation of full sources as required), followed by expert validation of pest presences with preference given to primary sources (Pasiecznik *et al.*, 2005). The CPC is a recent initiative, supported by over 40 technical, governmental, private-sector and nongovernmental partners, which utilizes similar standards for compiling and validating data, but includes additional data from sources such as the EPPO Pest Quarantine Register (PQR) and International Plant Protection Convention (IPPC) Official Pest Reports. The database is curated to retain only those records for which presence and correct identification of a pest can be assured with high confidence, supported by published sources. Presences are noted as widespread, restricted, rare or confined, where such information is available. We included all presences in our analysis, because in most instances abundance data were unavailable. The Plantwise database is the most comprehensive global database on crop pest distributions available, subsets of which have been used recently in national (Paini *et al.*, 2010) and global (Bebber *et al.*, 2013) analyses of pest distributions. The data are therefore likely to be suitable for a global analysis of observed pest distributions.

Records of pest presence in countries were available for, in order of number of species and pathovars: Fungi (419 species and pathovars), Coleoptera (219), Lepidoptera (252), Hemiptera (236), viruses (230), Bacteria (126), Nematoda (104), Diptera (110), Hymenoptera (26), Oomycota (59), Acari (55), Thysanoptera (34), and smaller numbers of Orthoptera, Isoptera, Gastropoda, various protists, Psocoptera, Neuroptera and Collembola. We list these groupings (which represent different taxonomic levels) for illustrative purposes. There were 60 907 records at national level.

Crop production and import data for 168 crops were obtained at national level for the decade 2001–2010 from the UN FAO (2013). For import, only unprocessed (fresh) crops were included in the analysis, as these are more likely to harbour living pests. The top 20 crops by mean annual production weight were (in decreasing order) sugar cane, maize, rice, wheat, potatoes, sugar beet, minor fresh vegetables, soybeans, cassava, oil palm fruit, barley, tomatoes, sweet potatoes, watermelons, bananas, cabbages and brassicas, grapes, onions, cotton seed and oranges. These 20 account for 81.5% of the total mean production weight.

Crop production data were used to calculate diversity indices of production. Diversity indices use population size estimates for the species in a community (Magurran, 2004), so in this case a pseudo-population size for each crop ‘species’ was derived from the mass of each crop produced, rounded to the nearest tonne. The commonly used Shannon index, and the rarefaction species richness (Hurlbert, 1971) for 1000-tonne samples, were calculated from recent average production of each crop in each country.

Two measures of country wealth were compared as predictors, mean *per capita* GDP from 2001 to 2010, and total wealth in 2005. *Per capita* GDP is correlated with scientific and technical capacity (Furman *et al.*, 2002) and has been used as a wealth indicator in previous studies of invasive species distributions (Taylor & Irwin, 2004; Essl *et al.*, 2011). However, the measure has been criticized in that it provides only a recent ‘snap-shot’ of a country's wealth rather than a long-term integration, and total wealth has been used as an aggregate of economic performance (Pyšek *et al.*, 2010). A disadvantage of total wealth is that estimates are available for only 152 countries, compared with 226 for *per capita* GDP. *Per capita* GDP (based on purchasing power parity) and total wealth data were obtained from the World Bank (2013) and IMF (2013). Mean expenditure on research and development (R&D) as a fraction of total GDP was included as another measure of scientific and technical capacity (data from World Bank, 2013). Several developing countries do not report R&D expenditure, and it was assumed that real expenditure was essentially zero. Because country wealth drives scientific and technological development, a direct measure of scientific output was also modelled. The number of scientific documents published per country in the fields of agriculture and biological sciences between 1996 and 2012 was obtained from the SCImago Journal & Country Rank database (Scimago Lab, 2013). We fitted models both including and excluding publication number, to determine the ultimate effects of economic variables and the proximate effects of scientific output on observed pest numbers.

The absolute latitude of the geographical centre of each country and its status – island, coastal or landlocked – were included as predictors. Because CABI historically supported agriculture in the Commonwealth, membership (historical and current) of the Commonwealth was also included. Nonagricultural transport was estimated by mean international tourist numbers per year, obtained from the World Bank (2013). Mean annual (1961–1990) precipitation was obtained from the TYN CY 1.1 dataset (Mitchell *et al.*, 2004). While latitude is an indicator of mean annual temperature and seasonality, mean annual precipitation is only moderately correlated with latitude (Supporting Information Table S1, Spearman's *r *=* *−0.54) and moisture could affect pest incidence independently of temperature. Spatial autocorrelation, due to the potential for spread of pests from neighbouring countries, was modelled for each country by the sum of unique pests for all countries sharing a land border.

The variance in numbers of pests per country was partitioned amongst biological, physical and socioeconomic predictors using linear models. Exploratory analysis using Generalized Additive Models indicated that nonlinear modelling was not required for the data (Figs S1, S2). The numbers of pests per country are count data, so square roots were taken when fitting the linear model to remove any dependence of the variance on the mean. Residuals were inspected for outliers and data with high leverage, and tested vs Normality using the Shapiro–Wilk test (Figs S3, S4).

Two models were fitted, differing by the inclusion of scientific publication number as a predictor. Model 1 was


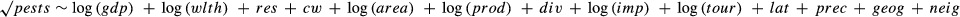


(*pests*, total number of reported pests per country; *gdp*, mean *per capita* GDP from 2001 to 2010; *wlth,* wealth in 2005; *res*, mean expenditure on research and development as a percentage of national GDP 2000–2009; *cw*, membership (past or current) of the Commonwealth; *area*, total land area of the country; *prod*, mean mass of crop production 2001–2010; *div*, a diversity index (either Shannon or the rarefaction species richness); *tour*, mean mass of agricultural imports of selected crops 2001–2010; *tour*, mean number of tourist visitors 2001–2010; *lat*, absolute latitude of the country centroid; *prec*, mean annual precipitation 1961–1990; *geog*, a geographical classification of a country as island, coastal or landlocked; and *neig*, total number of unique pests in neighbouring (shared land border) countries). Polynomials were fitted where this was indicated by exploratory plots. Three plausible interactions between predictors were tested: *gdp* × *res* because wealth should only influence observational capacity if some of that wealth is spent on research; *lat* × *prec* because, for example, warm, humid conditions could have different effects on pests than warm, dry conditions; and *gdp* × *geog* because exploratory analysis indicated that the number of pests per tonne of production increased more rapidly with *gdp* for islands than other geographical classes. Model 2 was fitted with the logarithm of the number of scientific publications (*sci*) as the first predictor.

Variables thought to be related to observational capability (*sci*, *gdp*, *wlth*, *res* and *cw*) were entered into the model first, so that *F*-tests on sequential sums-of-squares would test for the effect of physical variables (*area*, *prod*, *div*, *imp*, *tour*, *prec*, *geog*, *lat* and *neig*) only once variables that could indicate observational capacity had been taken into account. The model fit was examined to determine nonlinearity of responses and models with and without nonsignificant predictors were compared using an automated stepwise model selection procedure based on the Akaike Information Criterion, in order to determine the most appropriate model for the data (Venables & Ripley, 2002, p. 175). Residuals of fitted models were inspected for normality and leverage, and models re-fitted omitting outliers or countries with high leverage to determine their effect on the results (Tables S2,S3). No evidence of spatial autocorrelation in residuals was found (Fig. S5).

The competing trade (or ‘biological’) and observation (or ‘institutional’) hypotheses for the correlation of economic indicators with observed pest numbers per country could be differentiated by considering the likely ease of identification of different taxonomic pest groupings. Insects and other arthropods are likely to be the easiest groups both to detect and identify, normally requiring inspection of anatomical characters using the naked eye, a hand lens or microscope. For these organisms, even poorer countries are likely to have entomologists and taxonomists able to identify them, and the influence of *per capita* GDP and R&D expenditure on detection should be low. By contrast, microorganisms including fungi, bacteria, oomycetes and viruses, and soil-borne organisms such as nematodes, are harder to detect and often require modern molecular methods for positive identification. The availability of these techniques is likely to increase with wealth, and therefore the effect of wealth on the detection of microorganisms should be stronger than for arthropods. Conversely, if the effect of economic indicators is driven by historical trade, then there should be no difference among taxonomic groups in the effect, as there is no *a priori* reason to suspect that propagule pressure should increase at different rates with trade among the different taxonomic groups. To test these alternative hypotheses, Model 1 predictors were fitted for numbers of pests in different taxonomic groups, divided by the total number of pests in these groups, thereby giving the relative influence of economic factors to allow comparison among groups.

All analyses were conducted in R v3.0.1 (R Development Core Team, 2013). The function *stepAIC* in the package *MASS* was used for stepwise model selection. Maps were produced using the package *rworldmap*, and plots were produced using the packages *lattice* and *latticeExtra*.

## Results

Countries reported between 1 and 1200 pests (Fig. S6), with the largest numbers reported by the USA (1200), India (1063), China (1012), France (999) and Japan (973). The most widespread pests, in terms of number of countries in which they are found, were *Bemisia tabaci* (Insecta, present in 156 countries), *Aphis gossypii* (Insecta, 153 countries), *Planococcus citri* (Insecta, 143 countries), *Meloidogyne incognita* (Nematoda, 143 countries), *Agrius convolvuli* (Insecta, 141 countries), *Plutella xylostella* (Insecta, 138 countries), *Helicoverpa armigera* (Insecta, 135), *Myzus persicae* (Insecta, 133 countries), *Guignardia citricarpa* (Ascomycota, 131 countries), *Pseudocercospora angolensis* (Ascomycota, 131 countries) and *Nizara viridula* (Insecta, 131). Eight of these are insects. The numbers of pests in major functional groups (invertebrates, fungi and oomycetes, bacteria, viruses, nematoda and protozoans) were highly correlated (the first axis of a Principal Components Analysis on the square root of pest numbers in different classes explained 92.7% of variance among countries), that is countries with many fungal pests also have many viruses, etc.

Automated model selection for Model 1 determined that total wealth was the strongest predictor of observed pest numbers, explaining 71.2% of variance among countries, compared with 3.6% for *per capita* GDP when the latter was used as the wealth indicator. However, total wealth is highly correlated with country area and agricultural production (Table S1), and is therefore confounded with indicators of habitat area. By contrast, *per capita* GDP is weakly negatively correlated with area and production, whilst being positively correlated with wealth. Under the assumption that a country's wealth is, in part, based upon sovereign natural resources whose availability increases with land area, wealth per unit area and *per capita* GDP were compared as predictors. In this case, *per capita* GDP was retained in preference to wealth per unit area, demonstrating that, once confounding variables are controlled, *per capita* GDP is a better predictor of observed pest numbers than total wealth.

**Figure 1:**
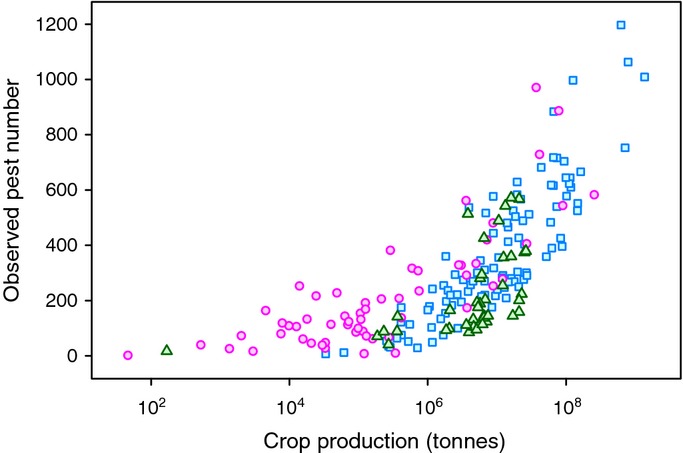
Observed pest number vs mean crop production 2001–2010. Islands, pink circles; coastal countries, blue squares; landlocked countries, green triangles.

**Figure 2:**
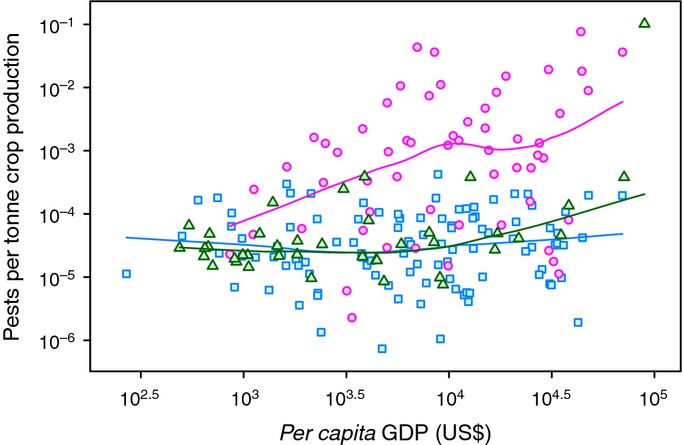
Pests per unit agricultural production vs *per capita* gross domestic product (GDP). Each point represents a country. Lines show cubic spline smooths to the log-transformed data. Pink circles (solid line), island nations; blue squares (dashed line), coastal nations; green triangles (dotted line), are landlocked nations. Pest numbers are scaled by production to facilitate cross-country comparisons. Islands generally report more pests and pathogens for a given level of production and *per capita* GDP.

In the final Model 1, using *per capita* GDP as the economic variable, the number of pests per country increased with *per capita* GDP, total crop production, expenditure on R&D, membership of the Commonwealth, precipitation and one measure of crop diversity, namely rarefaction species richness (Table 1). Crop production was the most important predictor (Fig. 1). Country area, tourism, total crop imports, absolute latitude and the number of pests in neighbouring countries were dropped from the model. No significant interactions were detected. Production was highly correlated with both land area and crop imports (Table S1), that is, large countries produce more food but also import more and, therefore, area and imports provided no additional information on observed pest numbers. Island nations reported more pests than coastal and landlocked nations (Table 1, Fig. 2), and the number of pests increased slightly with precipitation. Model 1 explained 86.9% of variance in observed pest numbers. Removal of outliers and influential data points had little effect on the model (Tables S2,S3).

**Table 1:** Model 1 of the square root of pest numbers per country, with *per capita* gross domestic product (GDP) as economic indicator

Predictor	Coefficient	Sum Sq.	df	Mean Sq.	*R*^2^	*F*	*P*
log_10_(*gdp*)	1.34 ± 0.47	296.3	1	296.3	3.4	48.0	< 10^−10^
*res*	3.69 ± 0.67	3111.3	2	1555.7	35.8	251.9	< 10^−15^
*res*^*2*^	−0.56 ± 0.16						
*cw*	1.8 ± 0.43	92.1	1	92.1	1.1	14.9	0.0002
log_10_(*prod*)	−1.82 ± 0.95	3636.7	2	1818.3	41.9	294.4	< 10^−15^
log_10_(*prod*)^2^	0.46 ± 0.08						
*div*^a^	0.12 ± 0.02	151.3	1	151.3	1.7	24.5	< 10^−5^
*prec*^b^	0.67 ± 0.25	149.7	1	149.7	1.7	24.2	< 10^−5^
*geog*	NA	111.9	2	56.0	1.3	9.1	0.0002
Coastal^c^	−3.76 ± 3.66						
Island-Costal	2.10 ± 0.57						
Landlocked-Costal	−0.68 ± 0.50						
Error	NA	1136.4	184	6.18	13.1	NA	NA
Model total	NA	7549.4	10	754.9	86.9	122.2	< 10^−15^
Total	NA	8685.8	194	NA	100.0	NA	NA

NA, not applicable.

Terms selected automatically using AIC. Terms are defined in the Materials and Methods section. Mean is the estimated coefficient. The sums of squares, degrees of freedom, mean square, coefficient of determination (*R*^2^), and *F*-tests are given for analysis of variance. Total model *R*^2^ = 86.9%.

a Rarefaction species richness.

b Precipitation in metres (not mm) to scale coefficient for presentation.

c The coefficient for Coastal nations is the intercept, that is coefficients for Island and Landlocked nations should be added to this when calculating expected values.

**Figure 3:**
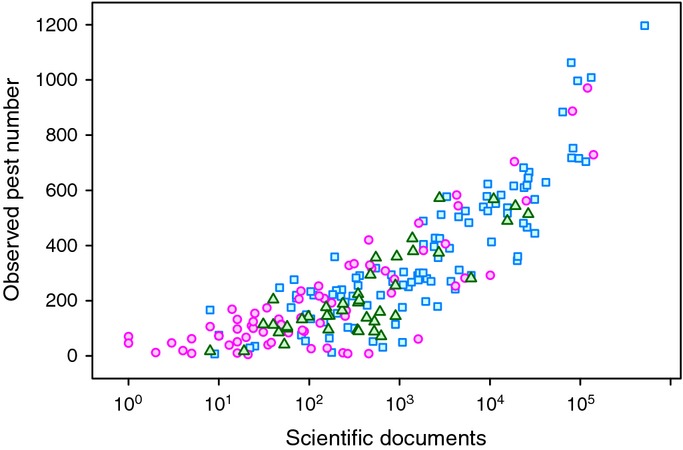
Observed pests vs scientific publications 1996–2012. Islands, pink circles; coastal countries, blue squares; landlocked countries, green triangles.

Automated model selection for Model 2 found that the number of scientific publications in agriculture and biological sciences was the most important predictor (*R*^2^ = 72.8%), followed by total crop production (Table 2, Fig. 3). R&D expenditure was not a significant predictor in Model 2. More science is published by large, wealthy countries that spend more on research, and also have greater agricultural production, crop diversity, crop imports and tourism (Table S1). The fractions of variance explained by Model 1 and Model 2 were approximately equal, and the predicted numbers of pests from the two models were highly correlated (*r *=* *0.98). Further analyses utilize Model 1, because the number of scientific publications is likely to be determined by ultimate causes such as country size, economic performance and expenditure on research.

**Table 2:** Model 2 of the square root of pest numbers per country, with log_10_ of number of scientific publications in agriculture and biological sciences as indicator of scientific capacity, and *per capita* gross domestic product (GDP) as economic indicator

Predictor	Mean	Sum Sq.	df	Mean Sq.	*R*^2^	*F*	*P*
log_10_(*sci* + 1)	−0.084 ± 0.70	6469.5	2	6469.5	75.2	644.1	< 10^−4^
log_10_(*sci* + 1)^2^	−0.55 ± 0.11						
*cw*	1.34 ± 0.38	96.9	1	96.9	1.1	19.3	< 10^−4^
log_10_(*prod*)	−0.83 ± 1.05	744.3	2	372.2	8.7	74.1	< 10^−4^
log_10_(*prod*)^2^	0.24 ± 0.09						
*div*^a^	0.10 ± 0.02	98.2	1	98.2	1.1	19.6	< 10^−4^
*prec*^b^	0.90 ± 0.22	180.0	1	180.0	2.1	35.9	< 10^−4^
*geog*	NA	63.9	2	32.0	0.7	6.4	0.0021
Coastal	0.96 ± 3.02						
Island-Coastal	1.76 ± 0.51						
Landlocked-Coastal	−0.03 ± 0.44						
Error	NA	949.2	189	5.0	11.0	NA	NA
Model total	NA	7652.8	9	850.3	89.0	134.0	< 10^−4^
Total	NA	8602.0	198	NA	100.0	NA	NA

Terms are defined in the Materials and Methods section. NA, not applicable.

Total model *R*^2^ = 89.0%.

a Rarefaction species richness.

b Precipitation in metres (not mm) to scale coefficient for presentation.

**Figure 4:**
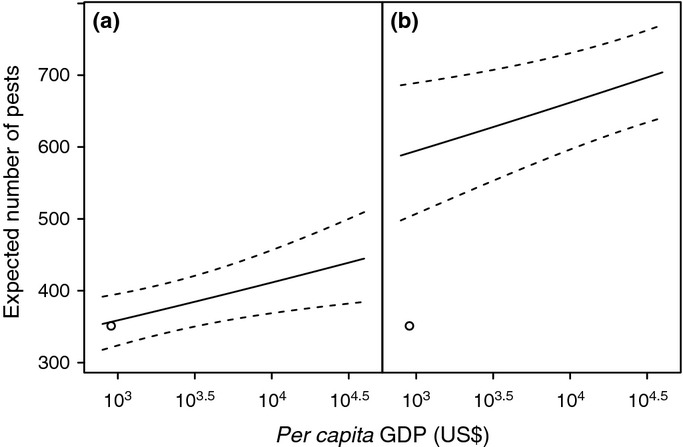
Effect of economic development on expected pest numbers reported by Myanmar. (a) Expected mean (solid line) and 95% confidence limits (dashed lines) for pest numbers vs *per capita* gross domestic product (GDP), with the current Myanmar-level investment in research and development (R&D) (0.11% of GDP). (b) Expected mean and 95% confidence limits for pest numbers vs *per capita* GDP, with US-level investment in R&D (2.64% of GDP). The circle in both panels shows the current reported pest number (351) and *per capita* GDP (US$904) for Myanmar.

The statistical requirements for transformations in the response and predictor variables make the model results rather cumbersome to interpret numerically, and therefore some examples of expected numbers of pests are given to illustrate the effects of varying production, *per capita* GDP and R&D expenditure. We take Myanmar as an example, given recent social changes that could yield rapid economic development. Myanmar is a coastal country with high amounts of agricultural production (mean 52 million tonnes, of which 54% is rice), but low *per capita* GDP (mean US $904 per annum, 2001–2010) and low investment in R&D (mean 0.11%). Three hundred and fifty-nine crop pests have been reported from Myanmar, with 362 (95% Confidence Limits 326–400) expected by the model. If a country such as the USA, with very high GDP (US $42 476 *per annum*) and high expenditure on R&D (2.64%), is effectively able to detect and report all of its crop pests, then the potential total number of agricultural pests in Myanmar can be estimated by fitting the model for Myanmar, but with US levels of GDP and R&D spending. This total is 723 (95% CI 657–793): thus, only around half of the possible total pest burden has been reported, if *per capita* GDP and R&D are solely indicative of observational capacity (Fig. 4). If GDP solely indicates invasion pressure, then realistic estimates of future GDP can indicate likely changes in pest numbers as a result. Myanmar could triple its *per capita* GDP by 2030 (Asian Development Bank, 2013), which would lead to a mean of 25 additional pests, in the absence of changes in other predictors.

**Figure 5:**
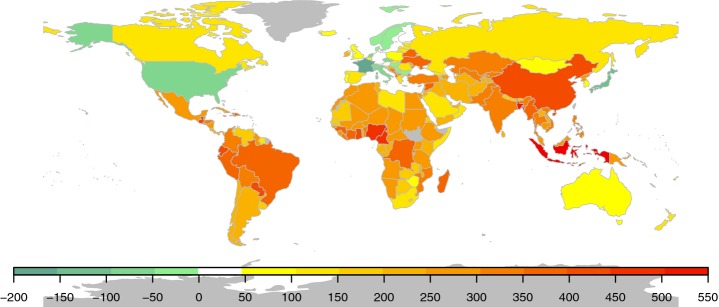
Expected additional number of pests per country. Expected numbers were predicted from the model, in which crop production and crop diversity were held at current levels, but *per capita* gross domestic product (GDP) and investment in research and development (R&D) were set to current USA levels. Grey shading denotes missing data for that country.

The model was used to predict the total number of pests expected in every country, if *per capita* GDP indicates observational capacity, and if the economic and technical power of the USA were available globally (Fig. S7). This resulted in a mean of 205 ± 9 additional pests expected per country (Fig. 5, Table S3). China (95% CI 1289–1552), India (1274–1538), Indonesia (1041–1274), the USA (1034–1228) and Brazil (1002–1222) have the largest expected number of pests. Given their high GDP and investment in research, developed nations in North America, Australasia and Western Europe were not expected to report many more pests. Indeed, Switzerland, France, the Netherlands and Japan have reported significantly more pests than the upper 95% confidence limit expected by the model under US-level *per capita* GDP and R&D. By contrast, many developing countries have reported far fewer pests than expected.

Scaling pest numbers by the total number of pests in each category to allow comparisons, the effect of *per capita* GDP on the fraction of all arthropods detected was less than half of the effect on micro-organisms, and the fraction of variance in arthropod numbers explained by *per capita* GDP was less than half of that explained in micro-organism numbers (Table 3). The effect of R&D was lowest for arthropods, but the fraction of variance explained was similar in arthropods, bacteria, fungi and oomycetes, and nematodes, but was larger for viruses.

**Table 3:** Effect of economic indicators on the numbers of pests in different taxonomic groups

	Effect size		*R*^2^			
*gdp*		*res*	*gdp*	*res*
Arthropoda	2.5 ± 1.0	2.9 ± 0.6	1.7	11.8
Bacteria	7.9 ± 1.6	3.5 ± 1.0	4.8	11.3
Fungi/Oomycota	6.8 ± 1.5	3.9 ± 1.0	3.5	12.7
Nematoda	5.2 ± 1.5	3.9 ± 1.0	3.6	11.1
Viruses	7.0 ± 1.3	4.9 ± 0.8	7.6	15.0

Terms are defined in the Materials and Methods section. Effect size is the linear model coefficient for pest numbers scaled as a percentage of the total number in the database. *R*^2^ is the coefficient of determination.

## Discussion

This study provides the first global assessment of the factors determining the known distributions of the world's crop pests and pathogens, covering 1901 crop-destroying organisms, and quantifying the influence of both socioeconomic and biophysical factors on our knowledge of pest distributions. Global patterns of pest species distributions differ significantly from wild species in being more diverse on islands but showing no latitudinal gradient, and are therefore similar to patterns detected for invasive species in general. The fraction of variance in pest diversity explained by the model is similar to that explained by land area and climate variables for wild species diversity (Hawkins *et al.*, 2003; Kalmar & Currie, 2006). Comparison among pest taxonomic groupings suggested that economic indicators such as *per capita* GDP are likely to be indicators of observational capacity, rather than solely indicators of trade. Assuming that *per capita* GDP is an indicator of observational capacity, the pest load of the developing world appears to be greatly underestimated, and this lack of knowledge may be severely hampering crop protection in some of the world's most important food-producing nations (Miller *et al.*, 2009; Flood, 2010; MacLeod *et al.*, 2010).

Countries are likely to vary greatly in their ability to detect and report pests. Model 2 demonstrated the importance of a proximate measure of scientific and technical capacity (publications in agricultural and biological sciences) in determining the number of pests recorded in the CABI databases. However, because the invasive species literature has interpreted economic variables as a measure of historical trade, and because economic status is an important driver of scientific capacity, we analysed the role of *per capita* GDP as a predictor of pest numbers. Because GDP increases with latitude (at least in recent times), relatively fewer species are reported from the tropics, an issue for other global species distribution databases (Yesson *et al.*, 2007; Boakes *et al.*, 2010; Feeley & Silman, 2011) and for invasive species (Pyšek *et al.*, 2008). *Per capita* GDP has been used in previous studies as a measure of scientific and technical capacity (Furman *et al.*, 2002), supplemented here by estimates of the fraction of total GDP invested in research and development. Modelling the influence of *per capita* GDP and R&D expenditure was used to control for this bias. In the biological invasions literature, GDP has been interpreted as a correlate of transportation infrastructure and trade, which facilitate invasions (but see Westphal *et al.*, 2008; Hulme, 2009). However, here the roles of habitat (crop production) and trade (imports and tourism) were fitted independently, and the role of GDP was therefore interpreted as an indicator of pest-detection capacity.

Plant pathologists have advocated the need for investment in pest monitoring and identification programmes (Miller *et al.*, 2009), but the effectiveness of expenditure on science has not hitherto been quantified. The model allowed the likely total numbers of pests, if countries had economies and technical capacity similar to the USA, to be estimated. This revealed that highly productive countries such as China, Brazil, India, Indonesia and the Philippines, are likely to be harbouring hundreds more crop pests than are currently known. These are growing economic powers, and it may be that, with sufficient investment in research, these missing pests will soon be revealed. Increasing numbers of invasive species are also expected (Ding *et al.*, 2008).

Total country wealth has been suggested as a better predictor of invasive species distributions than *per capita* GDP, because total wealth integrates long-term economic (and trade) activity (Pyšek *et al.*, 2010). However, total wealth is strongly correlated with total country area, and is therefore inadequate as an indicator of economic status unless appropriately scaled. Once wealth was corrected for area, *per capita* GDP was found to be a better predictor of pest presence. Additionally, the response of observed pest numbers per country to *per capita* GDP was greater in micro-organism pathogens compared with arthropod pests, supporting the idea that taxonomists in poorer countries can identify arthropods, but that greater technological capacity is required to positively identify micro-organisms.

Controlling for the ability of countries to detect and report pests enabled the effects of physical factors to be revealed. Crop production is the most important determinant of pest numbers per country. Production is analogous to the area of available habitat, and countries with more ‘habitat’ therefore support more pests, although it is possible that production also correlates with investment in crop protection. This suggests that increasing production will lead to greater numbers of pests. However, it must be acknowledged that the model contains no temporal component, and that model predictions are asymptotic values that say nothing of the time it would take for increases in production, or indeed GDP and investments in research, to influence the arrival and establishment of new pests, or the time taken for any newly trained plant pathologists to identify and report these new threats (Crooks, 2005).

The influence of diversity in agricultural production was equivocal, given that pest diversity increased with rarefaction species richness, but not with the Shannon diversity index. The two production diversity indicators are correlated (*r* = 0.77), so the reason for the difference is unclear. Most of the regions with the greatest crop diversity now (the Middle East, Mexico, Peru and China) are those where many of the world's crops were domesticated (Purugganan & Fuller, 2009), demonstrating a long-term persistence in the global distribution of crop diversity.

Greater pest reporting by island nations confirms some data for invasive species (but see Westphal *et al.*, 2008; Hulme, 2009). Island nations are generally smaller, wealthier, and invest less in research, than coastal and landlocked countries (Figs S1, S5). However, the effect of ‘islandness’ on pest detection is additional to these variables. Larger numbers of invasive species on islands have been linked to the importance of trade for island economies (Hulme, 2009), but islands do not import more crops than mainland countries, for a given amount of crop production (Fig. S2). This suggests that observational biases, rather than real differences in numbers of pests, are responsible. One possibility is that islands, some of which have been ravaged by introduced species in the past, invest disproportionately more in quarantine and customs than mainland nations, or are more intensively studied by researchers. Whether this is the case, and why coastal nations (which presumably also invest significantly in customs controls) are not differentiated from landlocked nations, is difficult to test empirically, given the lack of an obvious ‘rigour metric’ – current or historical – of countries' customs' officials.

Unlike many wild species, pest diversity did not show a latitudinal gradient, once latitudinal variation in factors such as *per capita* GDP had been taken into account. Precipitation has a significant, but weak, predictor of pest numbers. Numerous hypotheses have been proposed to explain the latitudinal gradient in species richness for natural ecosystems, including the greater availability of energy and water at the equator, the greater stability of the climate over short and long temporal scales, the larger area of suitable habitat and the greater likelihood that species ranges will overlap at the equator than at the poles (Hawkins *et al.*, 2003; Willig *et al.*, 2003; Krug *et al.*, 2009). If nonequilibrium mechanisms are important, whereby species diversity declines with latitude because migration from the equator has not yet taken place, then this could explain the lack of a latitudinal gradient for pests, because pest migrations are facilitated by human activities, thereby accelerating equilibration (Anderson *et al.*, 2004). If energy and water availability play a role, then the enhanced production rates of modern, mechanized agriculture at higher latitudes could support as many pests as are present in the tropics, contrary to the natural pattern where water and energy availability are greater at lower latitudes. Parasites and parasitoids of animals do not show consistent latitudinal patterns (Willig *et al.*, 2003), and it could be that crop pests are similar to these organisms in their responses.

Neither crop import volumes nor tourism are significant determinants of pest numbers, although human transport has been implicated in the spread of crop pests (Anderson *et al.*, 2004; Waage & Mumford, 2008) and invasive species (Westphal *et al.*, 2008). Because crop imports are highly correlated with crop production (perhaps counter-intuitively, countries that produce more food also import more food), any variation in pest diversity due to imports may be masked by including production in the model. A detailed analysis of trade matrices, investigating the potential movement of pests from exporter to importer countries for particular crops and their pests, could better reveal the role of trade.

This study presents the first comprehensive analysis of the global distributions of crop pests and pathogens, taking a synoptic approach to the total diversity of pests found in each country, and the physical and socioeconomic factors that influence this diversity. The role of economic factors, although similar to that found for other invasive species, has been interpreted as an observational bias, rather than an effect of trade. Partitioning the influence of these two drivers will require further study. The next step will be to undertake a more mechanistic analysis linking the known biology of pests (specifically, their host preferences) with the distributions of the crops, in order to determine how distributions vary among pest taxa and host plants, and whether the presence of individual pest species can be inferred in countries which have not reported occurrence.

## Supporting Information

Fig S1 Smooth terms from Generalized Additive Model, for covariates in the final version of Model 1. **Fig. S2** Smooth terms from Generalized Additive Model, for covariates in the final version of Model 2. **Fig. S3** Diagnostics for Model 1. **Fig. S4** Diagnostics for Model 2. **Fig. S5** Correlogram of model residuals. **Fig. S6** Observed pest numbers per country. **Fig. S7** Expected pest numbers per country. **Table S1** Correlations among covariates **Table S2** Coefficients and ANOVA for Model 1 with potential outliers and influential data removed **Table S3** Coefficients and ANOVA for Model 2 with potential outliers and influential data removed
