# Supplementary material for: Economic and physical determinants of the global distributions of crop pests and pathogens
Source: New Phytol. 2014 Feb 11;202(3):901–10. doi: 10.1111/nph.12722 (PMC4285859; doi:10.1111/nph.12722)
Supplement: Fig S1 — Smooth terms from Generalized Additive Model, for covariates in the final version of Model 1. Fig. S2 Smooth terms from Generalized Additive Model, for covariates in the final version of Model 2. Fig. S3 Diagnostics for Model 1. Fig. S4 Diagnostics for Model 2. Fig. S5 Correlogram of model residuals. Fig. S6 Observed pest numbers per country. Fig. S7 Expected pest numbers per country. Table S1 Correlations among covariates Table S2 Coefficients and ANOVA for Model 1 with potential outliers and influential data removed Table S3 Coefficients and ANOVA for Model 2 with potential outliers and influential data removed [file nph0202-0901-SD1.docx]

**Supporting Information Figs S1–S7 and Tables S1–S3**

**Economic and physical determinants of the global distributions of crop pests and pathogens**

Daniel P. Bebber, Timothy Holmes, David Smith & Sarah J. Gurr

**Supporting Information Figs S1-S7**

**Fig. S1. Smooth terms from Generalized Additive Model, for covariates in the final version of Model 1**. The dotted lines show 95% confidence limits of the smooths. The “rug” on the x-axis shows the location of the data points. The GAM explains 89.6 % of the deviance in the response. Fitted values are highly correlated with those of linear Model 1 (*r* = 0.99).

**Fig. S2. Smooth terms from Generalized Additive Model, for covariates in the final version of Model 2**. The dotted lines show 95% confidence limits of the smooths. The “rug” on the x-axis shows the location of the data points. The GAM explains 89.7 % of the deviance in the response. Fitted values are highly correlated with those of linear Model 2 (*r* = 0.99).

**Fig. S3. Diagnostics for Model 1**.

(a) Residuals vs fitted values, showing no relationship (smoothing spline shown in red) and no dependence of the variance on the mean.

(b) Normal quantile plot of Studentized residuals. The Shapiro-Wilk normality test did not reject the null hypothesis that the residuals came from a normal distribution (W = 0.99, *P* = 0.17) when all data included.

(c) Studentized residuals vs hat values, with area of circles proportional to Cook’s distance. Horizontal dashed lines at -2 and 2 can be used to indicate outliers, and vertical dashed line at twice the mean hat value can be used to indicate data with high leverage. Unusual data (with large residual, high leverage, or large Cook’s distance) are labelled: Israel (IL), PM (St. Pierre & Miquelon), East Timor (TL), French Polynesia (PF), New Caledonia (NC), Bermuda (BM) and Zimbabwe (ZW). Maximum Cook’s distance was for Bermuda (0.068). Fitted values from Model 1 with these countries omitted were almost identical (*r* = 0.9996) to fitted values from Model 1 with all data. Coefficients and ANOVA differed slightly, but not materially, from Model 1 with all data (Supporting Information Table S2).

**Fig. S4. Diagnostics for Model 2**.

(a) Residuals vs fitted values, showing no relationship (smoothing spline shown in red) and no dependence of the variance on the mean.

(b) Normal quantile plot of Studentized residuals. The Shapiro-Wilk normality rejected the null hypothesis that the data came from a normal distribution (W = 0.98, *P* = 0.02) when all data included, but did not reject when the data for French Polynesia (PF) were omitted (W = 0.995, *P* = 0.79).

(c) Studentized residuals vs hat values, with area of circles proportional to Cook’s distance. Horizontal dashed lines at -2 and 2 can be used to indicate outliers, and vertical dashed line at twice the mean hat value can be used to indicate data with high leverage. Unusual data (with large residual, high leverage, or large Cook’s distance) are labelled: Bulgaria (BG), Liechtenstein (LI), East Timor (TL), French Polynesia (PF), Niue (NU), Cayman Islands (KY), Nauru (NR). Fitted values from Model 2 with these countries omitted were almost identical (*r* = 0.9994) to fitted values from Model 2 with all data. Coefficients and ANOVA differed slightly, but not materially, from Model 2 with all data (Supporting Information Table S3).

**Fig. S5. Correlogram of model residuals.** Model 1 (blue line) and Model 2 (red line). Lag distances were calculated from centroid separations of different countries, and correlations calculated for country pairs falling into different lag ranges. There was no indication of spatial autocorrelation in the residuals.

**Fig. S6. Observed pest numbers per country.** Colour scale matches Fig. S7.

**Fig. S7. Expected pest numbers per country.** Predictions from the model (see main text) if all countries had USA levels of *per capita* GDP (US$ 42,476) and R & D expenditure (2.64 % of GDP).

**Supporting Informaton Tables S1–S3**

**Table S1. Correlations among covariates**. Predictors are transformed where this was required to linearize the relationship with the square root of crop pests per country.

|  | log_10_(*gdp*) | log_10_(*wlth*) | *res* | log_10_(*area*) | log_10_(*prod*) | *div* | log_10_(*imp*) | log_10_(*tour*+1) | *prec* | *lat* |
| --- | --- | --- | --- | --- | --- | --- | --- | --- | --- | --- |
| log_10_(*sci*+1) | 0.29 | 0.91 | 0.54 | 0.69 | 0.71 | 0.61 | 0.76 | 0.63 | -0.29 | 0.42 |
| log_10_(*gdp*) | - | 0.56 | 0.50 | -0.24 | -0.17 | -0.05 | 0.17 | 0.39 | -0.03 | 0.49 |
| log_10_(*wlth*) | - | - | 0.65 | 0.52 | 0.69 | 0.52 | 0.89 | 0.70 | -0.27 | 0.50 |
| *res* | - | - | - | 0.19 | 0.15 | 0.13 | 0.44 | 0.34 | -0.19 | 0.57 |
| log_10_(*area*) | - | - | - | - | 0.83 | 0.62 | 0.67 | 0.21 | -0.43 | 0.21 |
| log_10_(*prod*) | - | - | - | - | - | 0.72 | 0.74 | 0.41 | -0.31 | 0.19 |
| *div* | - | - | - | - | - | - | 0.67 | 0.38 | -0.42 | 0.20 |
| log_10_(*imp*) | - | - | - | - | - | - | - | 0.57 | -0.44 | 0.39 |
| log_10_(*tour*+1) | - | - | - | - | - | - | - | - | -0.13 | 0.26 |
| *prec* | - | - | - | - | - | - | - | - | - | -0.46 |

**Table S2. Coefficients and ANOVA for Model 1 with potential outliers and influential data removed**. The full model was re-fitted with all potential predictors, and the final model selected by forwards and backwards stepwise selection based on AIC. The omitted data (see Supporting Information Fig. S2c) were Bermuda, Israel, St. Pierre & Miquelon, East Timor, French Polynesia, New Caledonia and Zimbabwe. Total model *R*^2^ = 89.8 %.

| Predictor | Mean | Sum Sq. | df | Mean Sq. | *R*^2^ | *F* | *P* |
| --- | --- | --- | --- | --- | --- | --- | --- |
| log_10_(*gdp*) | 1.09 ± 0.43 | 402.4 | 1 | 402.4 | 5.4 | 88.9 | <10^-15^ |
| *res*  *res^2^* | 4.23 ± 0.79  -0.68 ± 0.25 | 2600.6 | 2 | 1300.3 | 34.7 | 287.4 | <10^-15^ |
| *cw* | 1.71 ± 0.40 | 46.1 | 1 | 46.1 | 0.6 | 10.2 | 0.0017 |
| log_10_(*prod*)  log_10_(*prod*)^2^ | -1.27 ± 1.15  0.40 ± 0.09 | 3261.0 | 2 | 1630.5 | 43.5 | 360.4 | <10^-15^ |
| *div^a^* | 0.18 ± 0.02 | 163.1 | 1 | 163.1 | 2.2 | 36.1 | <10^-7^ |
| *prec*^b^ | 0.85 ± 0.24 | 157.0 | 1 | 157.0 | 2.1 | 34.7 | <10^-7^ |
| *geog*  Coastal^c^  Island  Landlocked | NA  -4.61 ± 4.14  1.89 ± 0.53  -1.08 ± 0.45 | 98.2 | 2 | 49.1 | 1.3 | 10.9 | <10^-4^ |
| Error | NA | 764.7 | 169 | 4.52 | 10.2 | NA | NA |
| Model total | NA | 6728.5 | 10 | NA | 89.8 | 148.7 | <10^-15^ |
| Total | NA | 7493.2 | 179 | NA | 100.0 | NA | NA |

^a^ Rarefaction species richness.

^b^ Precipitation in metres (not mm) to scale coefficient for presentation.

^c^ The coefficient for Coastal nations is the intercept, i.e. coefficients for Island and Landlocked nations should be added to this when calculating expected values.

**Table S3. Coefficients and ANOVA for Model 2 with potential outliers and influential data removed**. The full model was re-fitted with all potential predictors, and the final model selected by forwards and backwards stepwise selection based on AIC. The omitted data (see Supporting Information Fig. S4c) were Bulgaria, Liechtenstein, East Timor, French Polynesia, Niue, Cayman Islands, and Nauru. Total model *R*^2^ = 90.3 %.

| Predictor | Mean | Sum Sq. | df | Mean Sq. | *R*^2^ | *F* | *P* |
| --- | --- | --- | --- | --- | --- | --- | --- |
| log_10_(*sci* + 1)  log_10_(*sci* + 1)^2^ | -0.21 ± 0.73  0.57 ± 0.12 | 5880.3 | 2 | 2940.2 | 75.8 | 709.1 | <10^-4^ |
| *cw* | 1.17 ± 0.36 | 84.9 | 1 | 84.9 | 1.1 | 20.5 | <10^-4^ |
| log_10_(*prod*)  log_10_(*prod*)^2^ | 0.31 ± 1.27  0.16 ± 0.10 | 665.0 | 2 | 332.5 | 8.6 | 80.2 | <10^-4^ |
| *div^a^* | 0.10 ± 0.02 | 96.4 | 1 | 96.4 | 1.2 | 23.2 | <10^-4^ |
| *prec*^b^ | 0.85 ± 0.02 | 179.8 | 1 | 179.8 | 2.3 | 43.4 | <10^-4^ |
| *geog*  Coastal  Island-Coastal  Landlocked-Coastal | NA  -2.45 ± 3.82  2.36 ± 0.49  -0.08 ± 0.41 | 101.2 | 2 | 50.6 | 1.3 | 12.2 | <10^-4^ |
| Error | NA | 754.6 | 182 | 4.2 | 9.7 | NA | NA |
| Model total | NA | 7007.6 | 9 | 778.6 | 90.3 | 187.8 | <10^-4^ |
| Total | NA | 7762.2 | 191 | NA | 100.0 | NA | NA |
